# Outcomes and Treatment Complications of Intravenous Urokinase Thrombolysis in Acute Ischemic Stroke in China

**DOI:** 10.3389/fneur.2021.685454

**Published:** 2021-07-12

**Authors:** Rongrong Zhang, Hui Wei, Yu Ren, Yanping Wu, Yetao Luo, Lei Zhang, Yingchao Huo, Jinzhou Feng, Philippe P. Monnier, Xinyue Qin

**Affiliations:** ^1^Department of Neurology, The First Affiliated Hospital of Chongqing Medical University, Chongqing, China; ^2^Department of Biostatistics, School of Public Health and Management, Chongqing Medical University, Chongqing, China; ^3^Donald K. Johnson Eye Institute, Krembil Research Institute, University Health Network, Toronto, ON, Canada; ^4^Department of Ophthalmology and Vision Science, Faculty of Medicine, University of Toronto, Toronto, ON, Canada; ^5^Department of Physiology, Faculty of Medicine, University of Toronto, Toronto, ON, Canada

**Keywords:** urokinase, alteplase, thrombolysis, ischemic stroke, treatment complications, outcomes

## Abstract

**Background:** Intravenous thrombolysis with alteplase benefits eligible patients with acute ischemic stroke. However, in some countries such as China, alteplase may be too expensive for low-income patients, and also for regions with low economic development. Urokinase is much less expensive than alteplase. This study aimed to assess the outcomes and treatment complications of urokinase in acute ischemic stroke patients, which are poorly understood.

**Methods:** This multicenter retrospective study included acute ischemic stroke patients who received intravenous urokinase or alteplase from January 2014 to January 2018 at 21 centers in China. Outcomes and treatment complications were analyzed by univariate and multivariate analyses.

**Results:** Among the 618 patients included in this study, 489 were treated with urokinase and 129 were treated with alteplase. Functional independence, no/minimal disability, mortality, intracranial hemorrhage (ICH), and symptomatic ICH did not significantly differ between the urokinase and alteplase groups in the univariate and multivariate analyses. However, the patients who received alteplase had a lower odds ratio (OR) of extracranial bleeding in the univariate analysis and a lower adjusted OR (aOR) in the multivariate analysis than the patients who received urokinase (OR = 0.410 [95% CI, 0.172–0.977], *p* = 0.038; aOR = 0.350 [95% CI, 0.144–0.854], *p* = 0.021). Furthermore, in patients treated with urokinase, the patients who received high-dose urokinase had a higher OR of extracranial bleeding in the univariate analysis and a higher aOR of extracranial bleeding in the multivariate analysis than patients who received low-dose urokinase (OR = 3.046 [95% CI, 1.696–5.470], *p* < 0.001; aOR = 3.074 [95% CI, 1.627–5.807], *p* = 0.001). Moreover, patients who received low-dose urokinase had similar outcomes and complications compared to patients treated with alteplase.

**Conclusions:** Patients treated with urokinase had similar outcomes but a higher risk of extracranial bleeding compared to patients treated with alteplase. The risk of extracranial bleeding was higher in the patients treated with high-dose urokinase than in the patients treated with low-dose urokinase. Patients who received low-dose urokinase had similar outcomes and complications compared to patients treated with alteplase. In countries such as China where some acute ischemic stroke patients cannot afford alteplase, urokinase may be a good alternative to alteplase for intravenous thrombolysis.

## Introduction

Ischemic stroke is a leading cause of death and disability worldwide. Theoretically, intravenous thrombolysis in the early hours after symptom onset can improve the chance of good recovery from ischemic stroke. Alteplase is the only intravenous thrombolytic drug that has been widely confirmed to improve functional outcomes for patients with acute ischemic stroke (1, 2). However, for low-income patients and those from regions with low economic development, alteplase is very expensive, resulting in heavy burdens on patients' families and society; the expense negatively impacts the enthusiasm for thrombolysis. Therefore, there is a pressing need to identify another safe and effective intravenous thrombolytic drug that is less expensive than alteplase.

Like alteplase, urokinase is a plasminogen activator. Urokinase has been approved by the China Food and Drug Administration and recommended in the latest Chinese stroke guidelines to use for intravenous thrombolysis in acute ischemic stroke patients (3). Supported by evidence from two trials with small samples (4, 5), the current Chinese stroke guidelines suggest that eligible acute ischemic stroke patients who are within 6 h of symptom onset can receive 1,000,000–1,500,000 IU intravenous urokinase thrombolysis. Urokinase is much less expensive than alteplase, at less than one-tenth the price. It is widely used in China, especially in regions with low economic development (6). However, the outcomes and treatment complications of intravenous urokinase thrombolysis in stroke patients remain unclear, given the small sample sizes and lack of an alteplase control group in previous urokinase studies (4, 5).

The objectives of this study were to characterize the outcomes and treatment complications in patients with acute ischemic stroke treated with intravenous urokinase within 6 h of symptom onset. We first compared the outcomes and treatment complications of urokinase and alteplase in acute ischemic stroke patients. Furthermore, we investigated the effect of the urokinase dose on outcomes and treatment complications.

## Methods

### Study Design and Patients

This multicenter retrospective study included patients from 21 participating centers (Supplementary Table 1) in China. Fifteen of these centers are located in regions with low economic development. All the centers have a neurology department; accordingly, these centers have certificated neurologists and neurology nurse specialists.

Supplementary Figure 1 shows a flow diagram of patient selection. The study population comprised patients with a final diagnosis of acute ischemic stroke who received intravenous urokinase or alteplase from January 2014 to January 2018. We excluded patients who did not have a complete medical history, those who were missing a National Institute of Health Stroke Scale (NIHSS) score before thrombolysis, those who were missing a modified Rankin Scale (mRS) score at 3 months, those with an onset-to-treatment time that was longer than 6 h or missing in the urokinase group, those with an onset-to-treatment time that was longer than 4.5 h or missing in the alteplase group, those with a dose that was other than 0.9 mg/kg or missing in the alteplase group, and those with a dose that was other than 1,000,000–1,500,000 IU or missing in the urokinase group.

### Measurements

The study population was described with respect to baseline demographics, vascular risk factors, NIHSS score before thrombolysis, and onset-to-treatment time. The outcomes were functional independence at 3 months (defined as an mRS score of 0–2 at 3 months), no/minimal disability at 3 months (defined as an mRS score of 0–1 at 3 months), and death within 3 months. The treatment complications analyzed comprised intracranial hemorrhage (ICH), symptomatic ICH, and extracranial bleeding. ICH was defined as any ICH on computed tomography (CT) scans between baseline and 7 days. Symptomatic ICH was defined according to the National Institute of Neurological Disorders and Stroke (NINDS) criteria as any ICH on CT scans combined with any decline in neurologic status (as measured by the NIHSS) between baseline and 7 days (7). According to the site of bleeding, extracranial bleeding was divided into gastrointestinal bleeding, subcutaneous hemorrhage, gingival bleeding, and other extracranial bleeding. Other extracranial bleeding included hematuria, subconjunctival hemorrhage, epistaxis, and subconjunctival hemorrhage. Major bleeding was defined as per the International Society of Thrombosis and Haemostasis as a decrease in hemoglobin level of 2 g/dL or requiring 2 or more units of packed red blood cells (8). All evaluations of imaging results and neurologic status were performed according to routine clinical practice at the local sites. The 3-month evaluations were conducted as outpatient consultations by clinicians who were highly experienced in outcome assessment. They were instructed not to access the medical records of the patient before the evaluation.

### Statistical Analysis

Firstly, patient characteristics were summarized. The variables are presented as count, percentage, or median (interquartile range [IQR]) as appropriate. We used the Mann-Whitney test to compare median data between the groups. Categorical variables were evaluated using the χ^2^ test. Secondly, univariable and multivariable logistic regression (Logit [Probability(C)] ~ Treatment + Covariates) was used to detect associations between treatment and outcomes/complications. In the equation, C represented outcomes/complications after treatment, and covariates included age, sex, previous transient ischemic attack or stroke, hypertension, diabetes mellitus, dyslipidemia, atrial fibrillation, history of smoking, and NIHSS score. The influencing factor of interest was the treatment, including intravenous urokinase and alteplase. Odds ratios (ORs) and corresponding 95% confidence intervals (CIs) were estimated accordingly, with patients who received urokinase being the reference group. Thirdly, subgroup analyses were performed. We focused on patients who received different urokinase dose (1,000,000 IU vs. 1,200,000-1,500,000 IU) using logistic regression above, with patients who received low-dose urokinase (1,000,000 IU) is the reference group. Besides, we focused on patients who received low-dose urokinase or alteplase using logistic regression above, with patients who received low-dose urokinase (1,000,000 IU) is the reference group. All analyses were performed using SAS v.9.4 (SAS Institute, Cary, NC, USA). Values of *p* < 0.05 were regarded as statistically significant.

## Results

Across the 21 participating centers, 597 patients with acute ischemic stroke received intravenous urokinase and 150 received intravenous alteplase between January 2014 and January 2018. Among these patients, 489 treated with urokinase and 129 treated with alteplase met the inclusion criteria (Supplementary Figure 1). The baseline clinical characteristics of the patients are described in Table 1. The onset-to-treatment time differed between the urokinase and alteplase groups (222 vs. 162 min, *p* < 0.001). Otherwise, the two groups were balanced.

**Table 1:** Baseline clinical characteristics of patients treated with urokinase or alteplase.

**Variable**	**Urokinase (*n* = 489)**	**Alteplase (*n* = 129)**	***P*-value**
Age, year, median (IQR)	69 (61–77)	71 (60–76.5)	0.717
Female, *n* (%)	205 (41.9)	56 (43.4)	0.761
Previous TIA or stroke, *n* (%)	97 (19.8)	32 (24.8)	0.217
Hypertension, *n* (%)	253 (51.7)	68 (52.7)	0.844
Diabetes mellitus, *n* (%)	98 (20.0)	28 (21.7)	0.676
Dyslipidemia, *n* (%)	102 (20.9)	33 (25.6)	0.248
Atrial fibrillation, *n* (%)	131 (26.8)	31 (24.0)	0.526
Any history of smoking, *n* (%)	167 (34.2)	48 (37.2)	0.517
Pre-thrombolysis systolic blood pressure, mm Hg, median (IQR)	155 (138–166)	157 (138–173)	0.082
Pre-thrombolysis diastolic blood pressure, mm Hg, median (IQR)	85 (78–93)	87 (79–93)	0.463
Pre-thrombolysis blood glucose, mmol/L, median (IQR)	7.4 (6.1–10.5)	7.3 (6.2–10.9)	0.791
NIHSS score, median (IQR)	10 (5–15)	10 (6–15)	0.943
Onset-to-treatment time, min, median (IQR)	222 (162–294)	162 (123–207)	<0.001

*IQR, interquartile range; NIHSS, National Institutes of Health Stroke Scale; and TIA, transient ischemic attack*.

**Figure 1:**
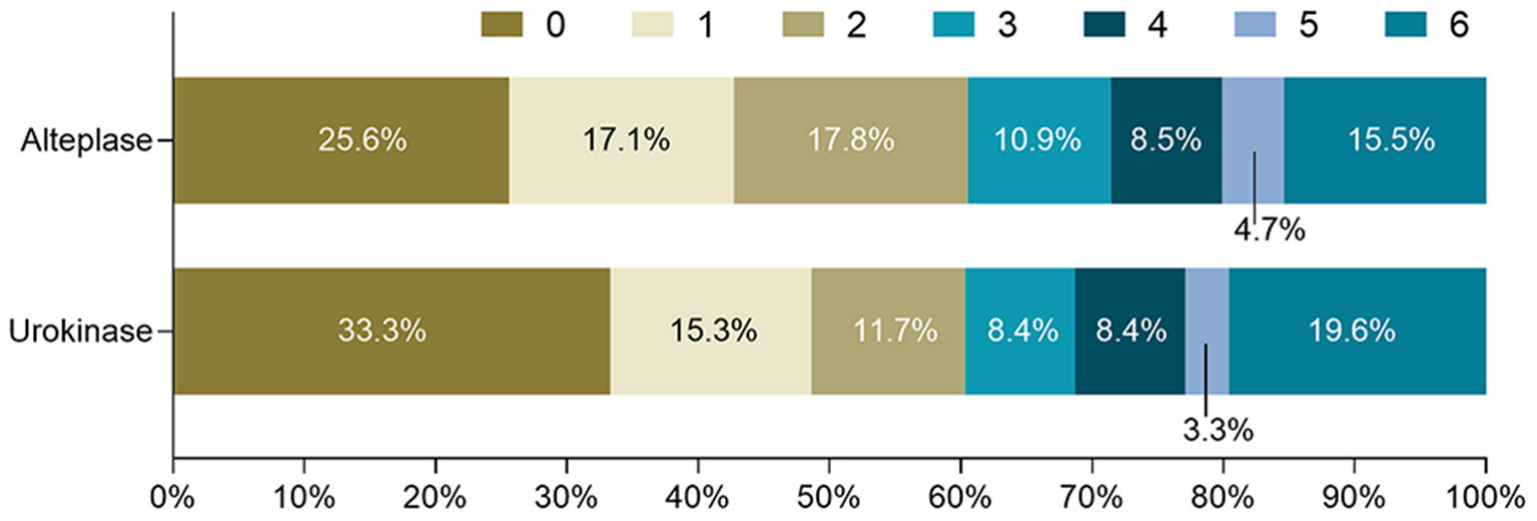
Distribution of mRS scores at 3 months by thrombolytic drug. mRS, modified Rankin Scale.

In Table 2, outcomes and treatment complications are compared between the urokinase and alteplase groups. The rates of functional independence and no/minimal disability were similar between the two groups in the univariate and multivariate analyses. Figure 1 shows the distributions of mRS scores at 3 months by thrombolytic drug. Similarly, mortality, ICH, and symptomatic ICH did not significantly differ between the urokinase and alteplase groups in the univariate and multivariate analyses. However, compared to patients who received urokinase, patients who received alteplase had a lower OR of extracranial bleeding in the univariate analysis (OR = 0.410 [95% CI, 0.172–0.977], *p* = 0.038) and a lower adjusted OR (aOR) in the multivariate analysis (aOR= 0.350 [95% CI, 0.144–0.854], *p* = 0.021). We further analyzed the extracranial bleeding according to the site of bleeding. Gastrointestinal bleeding, subcutaneous hemorrhage, gingival bleeding, and other extracranial bleeding did not significantly differ between the urokinase and alteplase groups in the univariate and multivariate analyses. Moreover, regarding major extracranial bleeding, there was also no significant difference between the two groups.

**Table 2:** Outcomes and treatment complications in patients treated with urokinase or alteplase.

**Outcome**	**Urokinase (*n* = 489)**	**Alteplase (*n* = 129)**	**OR^a^ (95% CI)**	***P*-value**	**aOR^b^ (95% CI)**	***P*-value**
Functional independence at 3 months, *n* (%)	295 (60.3)	78 (60.5)	1.006 (0.676–1.496)	0.977	1.012 (0.634–1.616)	0.960
No/minimal disability at 3 months, *n* (%)	238 (48.7)	55 (42.6)	0.784 (0.530–1.159)	0.222	0.726 (0.456–1.154)	0.176
Death within 3 months, *n* (%)	96 (19.6)	20 (15.5)	0.751 (0.444–1.272)	0.285	0.656 (0.356–1.209)	0.176
ICH, *n* (%)	47 (9.6)	11 (8.5)	0.877 (0.441–1.743)	0.707	0.778 (0.366–1.653)	0.513
Symptomatic ICH, *n* (%)	20 (4.1)	6 (4.7)	1.144 (0.450-2.910)	0.778	0.730 (0.243–2.191)	0.575
Extracranial bleeding, *n* (%)	52 (10.6)	6 (4.7)	0.410 (0.172–0.977)	0.038	0.350 (0.144–0.854)	0.021
Gastrointestinal bleeding, *n* (%)	21 (4.3)	2 (1.6)	0.351 (0.081–1.517)	0.229	0.245 (0.054–1.120)	0.070
Subcutaneous hemorrhage, *n* (%)	18 (3.7)	2 (1.6)	0.412 (0.094–1.799)	0.349	0.363 (0.081–1.634)	0.187
Gingival bleeding, *n* (%)	15 (3.1)	2 (1.6)	0.498 (0.112–2.204)	0.526	0.485 (0.107–2.191)	0.347
Other extracranial bleeding, *n* (%)	6 (1.2)	1 (0.8)	0.629 (0.075–5.271)	1.000	0.480 (0.054–4.245)	0.509
Major extracranial bleeding, *n* (%)	7 (1.4)	1 (0.8)	0.538 (0.066–4.412)	0.882	0.459 (0.046–4.614)	0.508

*aOR, adjusted odds ratio; ICH, intracerebral hemorrhage; and OR, odds ratio*.

a *Urokinase is the reference group*.

b *Urokinase is the reference group. Multivariate analysis was adjusted for age, sex, previous transient ischemic attack or stroke, hypertension, diabetes mellitus, dyslipidemia, atrial fibrillation, history of smoking, and National Institutes of Health Stroke Scale (NIHSS) score*.

Furthermore, we investigated the effect of urokinase dose on outcomes and treatment complications in patients treated with urokinase. Regarding the urokinase dose, 355 (72.6%) patients were administered 1,000,000 IU (low-dose group), and 134 (27.4%) patients were administered 1,200,000–1,500,000 IU (high-dose group). No patients received 1,000,001–1,199,999 IU urokinase in our study. Outcomes and treatment complications were compared between the low- and high-dose urokinase groups (Table 3). The rates of functional independence and no/minimal disability were similar between the two groups in the univariate and multivariate analyses. Similarly, mortality, ICH, and symptomatic ICH did not differ significantly between the low- and high-dose groups in the univariate and multivariate analyses. However, in comparison to low-dose urokinase, patients who received high-dose urokinase were more likely to have extracranial bleeding (7.3 vs. 19.4%; OR = 3.046 [95% CI, 1.696–5.470], *p* < 0.001; aOR = 3.074 [95% CI, 1.627–5.807], *p* = 0.001). Regarding the site of extracranial bleeding, rates of gastrointestinal bleeding (2.8 vs. 8.2%; OR = 3.085 [95% CI, 1.279–7.444], *p* = 0.009; aOR = 4.020 [95% CI, 1.484–10.892], *p* = 0.006) and subcutaneous hemorrhage (2.0 vs. 8.2%; OR = 4.446 [95% CI, 1.686–11.724], *p* = 0.003; aOR = 3.612 [95% CI, 1.278–10.208], *p* = 0.015) were significantly increased in the high-dose group. Furthermore, patients who received high-dose urokinase had a similar rate of major extracranial bleeding in the univariate analysis but a higher aOR in the multivariate analysis compared to patients who received low-dose urokinase (0.8 vs. 3.0%; OR = 3.610 [95% CI, 0.797–16.349], *p* = 0.177; aOR = 9.739 [95% CI, 1.650–57.468], *p* = 0.012).

**Table 3:** Outcomes and treatment complications by urokinase dose.

**Outcome**	**Urokinase dose**		**OR^a^ (95% CI)**	***P-*value**	**aOR^b^ (95% CI)**	***P*-value**
	**1,000,000 IU, *n* = 355**	**1,200,000-1,500,000 IU, *n* = 134**				
Functional independence at 3 months, *n* (%)	212 (59.7)	83 (61.9)	1.098 (0.730–1.651)	0.654	0.885 (0.534–1.464)	0.634
No/minimal disability at 3 months, *n* (%)	169 (47.6)	69 (51.5)	1.168 (0.785–1.739)	0.443	1.079 (0.647–1.799)	0.771
Death, *n* (%)	72 (20.3)	24 (17.9)	0.858 (0.514–1.431)	0.556	1.175 (0.638–2.164)	0.604
Symptomatic ICH, *n* (%)	14 (3.9)	6 (4.5)	1.142 (0.429–3.035)	0.790	1.818 (0.554–5.969)	0.324
ICH, n (%)	35 (9.9)	12 (9.0)	0.899 (0.452–1.789)	0.762	1.132 (0.516–2.487)	0.757
Extracranial bleeding, *n* (%)	26 (7.3)	26 (19.4)	3.046 (1.696–5.470)	<0.001	3.074 (1.627–5.807)	0.001
Gastrointestinal bleeding, *n* (%)	10 (2.8)	11 (8.2)	3.085 (1.279–7.444)	0.009	4.020 (1.484–10.892)	0.006
Subcutaneous hemorrhage, *n* (%)	7 (2.0)	11 (8.2)	4.446 (1.686–11.724)	0.003	3.612 (1.278–10.208)	0.015
Gingival bleeding, *n* (%)	9 (2.5)	6 (4.5)	1.802 (0.629–5.164)	0.414	2.262 (0.723–7.074)	0.161
Other extracranial bleeding, *n* (%)	4 (1.1)	2 (1.5)	1.330 (0.241–7.345)	1.000	1.761 (0.255–12.150)	0.566
Major extracranial bleeding, *n* (%)	3 (0.8)	4 (3.0)	3.610 (0.797–16.349)	0.177	9.739 (1.650–57.468)	0.012

*aOR, adjusted odds ratio; CI, confidence interval; ICH, intracerebral hemorrhage; and OR, odds ratio*.

a *Low-dose (1,000,000 IU) is the reference group*.

b *Low-dose (1,000,000 IU) is the reference group. Multivariate analysis was adjusted for age, sex, previous transient ischemic attack or stroke, hypertension, diabetes mellitus, dyslipidemia, atrial fibrillation, history of smoking, and National Institutes of Health Stroke Scale (NIHSS) score*.

Moreover, we conducted a subgroup analysis of low-dose urokinase and alteplase. Outcomes and treatment complications were compared between the low-dose urokinase group and the alteplase group (Table 4). Functional independence, no/minimal disability, mortality, ICH, symptomatic ICH, and extracranial bleeding were similar between the two groups in the univariate and multivariate analyses.

**Table 4:** Subgroup analysis of low-dose urokinase and alteplase.

**Outcome**	**Low-dose urokinase (*n* = 355)**	**Alteplase (*n* = 129)**	**OR^a^ (95% CI)**	***P*-value**	**aOR^b^ (95% CI)**	***P*-value**
Functional independence at 3 months, *n* (%)	212 (59.7)	78 (60.5)	1.032 (0.683–1.557)	0.882	1.002 (0.612–1.639)	0.995
No/minimal disability at 3 months, *n* (%)	169 (47.6)	55 (42.6)	0.818 (0.545–1.228)	0.332	0.714 (0.438–1.165)	0.177
Death within 3 months, *n* (%)	72 (20.3)	20 (15.5)	0.721 (0.419–1.241)	0.236	0.666 (0.353–1.257)	0.209
ICH, *n* (%)	35 (9.9)	11 (8.5)	0.852 (0.419–1.733)	0.659	0.765 (0.346–1.693)	0.509
Symptomatic ICH, *n* (%)	14 (3.9)	6 (4.7)	1.188 (0.447–3.161)	0.730	0.912 (0.281–2.959)	0.878
Extracranial bleeding, *n* (%)	26 (7.3)	6 (4.7)	0.617 (0.248–1.536)	0.295	0.564 (0.220–1.444)	0.232
Gastrointestinal bleeding, *n* (%)	10 (2.8)	2 (1.6)	0.543 (0.117–2.513)	0.644	0.589 (0.119–2.908)	0.516
Subcutaneous hemorrhage, *n* (%)	7 (2.0)	2 (1.6)	0.783 (0.161–3.818)	1.000	0.758 (0.148–3.893)	0.740
Gingival bleeding, *n* (%)	9 (2.5)	2 (1.6)	0.605 (0.129–2.840)	0.766	0.572 (0.117–2.799)	0.490
Other extracranial bleeding, *n* (%)	4 (1.1)	1 (0.8)	0.686 (0.076–6.191)	1.000	0.526 (0.054–5.145)	0.581
Major extracranial bleeding, *n* (%)	3 (0.8)	1 (0.8)	0.917 (0.094–8.892)	1.000	0.972 (0.063–15.047)	0.984

*aOR, adjusted odds ratio; CI, confidence interval; ICH, intracerebral hemorrhage; and OR, odds ratio*.

a *Low-dose urokinase is the reference group*.

b *Low-dose urokinase is the reference group. Multivariate analysis was adjusted for age, sex, previous transient ischemic attack or stroke, hypertension, diabetes mellitus, dyslipidemia, atrial fibrillation, history of smoking, and National Institutes of Health Stroke Scale (NIHSS) score*.

## Discussion

To the best of our knowledge, the present study is the first to compare the outcomes and treatment complications associated with 1,000,000–1,500,000 IU urokinase administered within 6 h from stroke onset and 0.9 mg/kg alteplase administered within 4.5 h from stroke onset in acute ischemic stroke patients. Furthermore, this is also the largest report of ischemic stroke patients treated with intravenous urokinase. We showed that patients treated with urokinase had similar outcomes but a higher risk of extracranial bleeding compared to patients treated with alteplase. Furthermore, patients treated with low-dose urokinase (1,000,000 IU) had a similar functional outcome but a lower risk of extracranial bleeding compared to patients treated with high-dose urokinase (1,200,000–1,500,000 IU). Moreover, patients received low-dose urokinase had similar outcomes and complications compared to patients treated with alteplase.

In recent decades, thrombolytic therapy for acute ischemic stroke has been extensively explored. Intravenous thrombolysis using 0.9 mg/kg alteplase administered within 4.5 h from symptom onset has been widely confirmed to benefit eligible patients with acute ischemic stroke and recommended by the latest international stroke guidelines (2, 3). Unlike patients treated with alteplase, patients treated with streptokinase, another thrombolytic drug, have a high mortality and symptomatic ICH rate. In patients treated with streptokinase in the Australian Streptokinase (ASK) Trial (9) and Multicenter Acute Stroke Trial-Europe (MAST-E) (10), the mortality was 33.6% to 44.9% and the symptomatic ICH rate was 19.0% to 21.2%. Furthermore, desmoteplase did not improve functional outcomes in the Desmoteplase in Acute Ischemic Stroke 2 (DIAS-2) (11) or Desmoteplase in Acute Ischemic Stroke 3 (DIAS-3) trials (12), with 51.3% of patients who received desmoteplase and 49.8% of patients who received placebo achieving functional independence at 3 months in DIAS-3 (12). Although reliable clinical evidence is limited, urokinase has been widely used in China and recommended in the Chinese stroke guidelines (3). Chinese stroke guidelines recommend 1,000,000–1,500,000 IU urokinase thrombolysis based on the results of two trials with small samples conducted two decades ago (4, 5). The first study was an open-label pilot clinical trial. All of the 409 enrolled ischemic stroke patients received 50,000–1,500,000 IU urokinase. In this trial, 46.6% of patients had European Stroke Scale (ESS) scores ≥ 95 at 3 months after stroke onset and 3.9% had symptomatic ICH (4). The second study was a multicenter, double-blind, placebo-controlled randomized clinical trial (RCT). The trial enrolled 465 ischemic stroke patients, of whom 155 were randomly assigned to the 1,500,000 IU urokinase group, 162 to the 1,000,000 IU urokinase group, and 148 to the placebo group. At 3 months after stroke onset, the mRS scores were significantly lower in the 1,500,000 and 1,000,000 IU urokinase group than the placebo group. The symptomatic ICH rates were similar among the three groups (5). Recently, several comparative studies on the efficacy and safety of alteplase and urokinase for treating acute ischemic stroke have been conducted (13–15). These studies all demonstrated that urokinase and alteplase have similar therapeutic effects. However, the bleeding risks were different. Sun et al. reported that the ICH risk of alteplase was lower than urokinase (14). Bao et al. found that patients treated with urokinase had a lower frequency of bleeding (13). Wang et al. reported that the rates of subcutaneous ecchymosis, gingival bleeding, and ICH were similar between urokinase and alteplase (15). It should be noted that in these studies, the onset-to-treatment time for alteplase was not 4.5 h from symptom onset or the alteplase dose was not 0.9 mg/kg. Furthermore, the sample sizes in these studies were small, and the clinical data collected were limited, i.e., lack of mRS scores.

Among acute ischemic stroke patients treated with intravenous alteplase thrombolysis in the Norwegian Tenecteplase Stroke Trial (NOR-TEST) (16), NINDS (7), European Cooperative Acute Stroke Study III (ECASS III) (17), and Enhanced Control of Hypertension and Thrombolysis Stroke Study (ENCHANTED) (18), 39.2–62.6% had no/minimal disability at 3 months (7, 16–18), and 57.3–78.4% had functional independence at 3 months (16–18). In patients treated with urokinase in the prior RCT urokinase study, the rate of no/minimal disability at 3 months was 44.9% in the 1,500,000 IU urokinase group and 45.5% in the 1,000,000 IU urokinase group, while the no/minimal disability rate in the placebo group was 31.9% (5). In the present study, 48.7 and 42.6% of the patients who received urokinase and alteplase, respectively, had no/minimal disability at 3 months. The rates of functional independence at 3 months were 60.3 and 60.5% in patients who received urokinase and alteplase, respectively. Thus, 1,000,000–1,500,000 IU urokinase administered within 6 h from stroke onset appeared to be at least as effective in achieving a good clinical outcome as 0.9 mg/kg alteplase administered within 4.5 h.

In prior alteplase clinical trials, the mortality of patients who received alteplase was 4.7% to 18.8% (7, 16–18). In prior urokinase trials, the mortality of patients who received urokinase was 10.7–12.2% (4, 5). In the current study, the mortality rate was 19.6% in the urokinase group and 15.5% in the alteplase group, with no significant difference between the two groups. Furthermore, we also did not detect a difference in ICH or symptomatic ICH rates between the urokinase and alteplase groups. Symptomatic ICH was noted in 4.2% of patients in our urokinase group according to the NINDS criteria (7). In the prior urokinase RCT, the rate of symptomatic ICH among patients who received urokinase (1,000,000 or 1,500,000 IU) was 3.8%, which is similar to our study; however, the definition of symptomatic ICH used in the prior RCT was unclear (5). In our study, compared to patients treated with alteplase, patients treated with urokinase exhibited an increased risk of extracranial bleeding. Unlike alteplase, which activates plasminogen only around the thrombus to lyse it with minimal activation of the circulating plasminogen, urokinase activates plasminogen both around the clot and in the circulating plasma, which results in an increased risk of extracranial bleeding (19).

The Chinese stroke guidelines recommend 1,000,000–1,500,000 IU urokinase for thrombolytic therapy as safe and effective (3). However, the guidelines do not state how to determine the patient-specific dose within 1,000,000–1,500,000 IU. Therefore, the specific urokinase dose of each patient was determined by the patients' treating doctors. The decision is typically based on each patient's weight and risk of bleeding. Given the lack of evidence on the bleeding risk of urokinase thrombolysis, the doctors assessed the bleeding risk in accordance with the bleeding risk assessment related to alteplase thrombolysis. In the current study, there were no significant differences in the rates of good outcomes, mortality, symptomatic ICH, or any ICH between the low- (1,000,000 IU) and high-dose (1,200,000–1,500,000 IU) urokinase groups. These findings are consistent with the prior RCT in which the outcome and complication rates were similar between the patients who received 1,000,000 or 1,500,000 IU urokinase (5). However, in our study, compared to the low-dose group, patients treated with high-dose urokinase had an increased risk of extracranial bleeding, which was not investigated in the prior RCT. Furthermore, we conducted a subgroup analysis of low-dose urokinase and alteplase. Outcomes and treatment complications were similar between the two groups.

Our study has several limitations. An important limitation is the retrospective design. The 3-month evaluations were not truly measured by blinded assessors; instead, treating physicians were relied upon to not access patient data before the assessments. Furthermore, the number of patients who received alteplase was lower than the number of patients who received urokinase. Most of our participating centers are located in regions with low economic development, where a large proportion of patients could not afford alteplase or the required onset-to-treatment time for alteplase treatment was exceeded. Furthermore, the patient records were much less complete than we expected. As a result, the information we were able to analyze in our study was limited. For instance, we could not analyze urokinase dose based on patient weight as many of the patient records did not include weight.

In conclusion, our findings indicate that patients treated with urokinase have similar outcomes but an increased risk of extracranial bleeding compared to patients treated with alteplase. Furthermore, the risk of extracranial bleeding was increased in patients treated with high-dose urokinase compared to patients treated with low-dose urokinase. Moreover, patients who received low-dose urokinase had similar outcomes and complications compared to patients treated with alteplase. For acute ischemic stroke patients who cannot afford alteplase, urokinase may be a good choice for intravenous thrombolysis.

## Data Availability Statement

The raw data supporting the conclusions of this article will be made available by the authors, without undue reservation.

## Ethics Statement

The studies involving human participants were reviewed and approved by the Institutional Review Board of the First Affiliated Hospital of Chongqing Medical University. Written informed consent was obtained from all participants for their participation in this study.

## Author Contributions

XQ, RZ, and HW conceptualized this work. HW, YR, YW, LZ, and YH collected the data. YL and RZ performed the statistical analysis. XQ supervised the study. RZ and XQ prepared the manuscript. JF and PM revised the manuscript. All authors have read and approved the manuscript.

## Conflict of Interest

The authors declare that the research was conducted in the absence of any commercial or financial relationships that could be construed as a potential conflict of interest.
