# Supplementary material for: Outcomes and Treatment Complications of Intravenous Urokinase Thrombolysis in Acute Ischemic Stroke in China
Source: Front Neurol. 2021 Jul 12;12:685454. doi: 10.3389/fneur.2021.685454 (PMC8311518; doi:10.3389/fneur.2021.685454)
Supplement: Supplementary file 1 [file Table_1.DOCX]

Supplementary Material

**Supplementary Table 1.** Enrolled centers.

| **Centers** |
| --- |
| The First Affiliated Hospital of Chongqing Medical University |
| The People’s Hospital of Tongliang District |
| The People’s Hospital of Bishan District |
| Chongqing Iron and Steel General Hospital |
| The Dazu District People’s Hospital |
| Chongqing General Hospital |
| Chongqing Sanbo Changan Hospital |
| Chongqing Emergency Medical Center |
| The People’s Hospital of Rongchang District |
| Zhongxian Hospital of Chongqing |
| Yongchuan Hospital of Chongqing Medical University |
| Chongqing Qijiang People’s Hospital |
| The Ninth People’s Hospital of Chongqing |
| The First People’s Hospital of Chongqing Liang Jiang New Area |
| Huaxi Hospital |
| Banan People’s Hospital of Chongqing |
| The First Hospital of Yibin |
| Dazhu County People’s Hospital |
| Hejiang People’s Hospital |
| People’s Hospital of Luxian County |
| The Affiliated Hospital of Southwest Medical University |

# Supplementary Figure 1


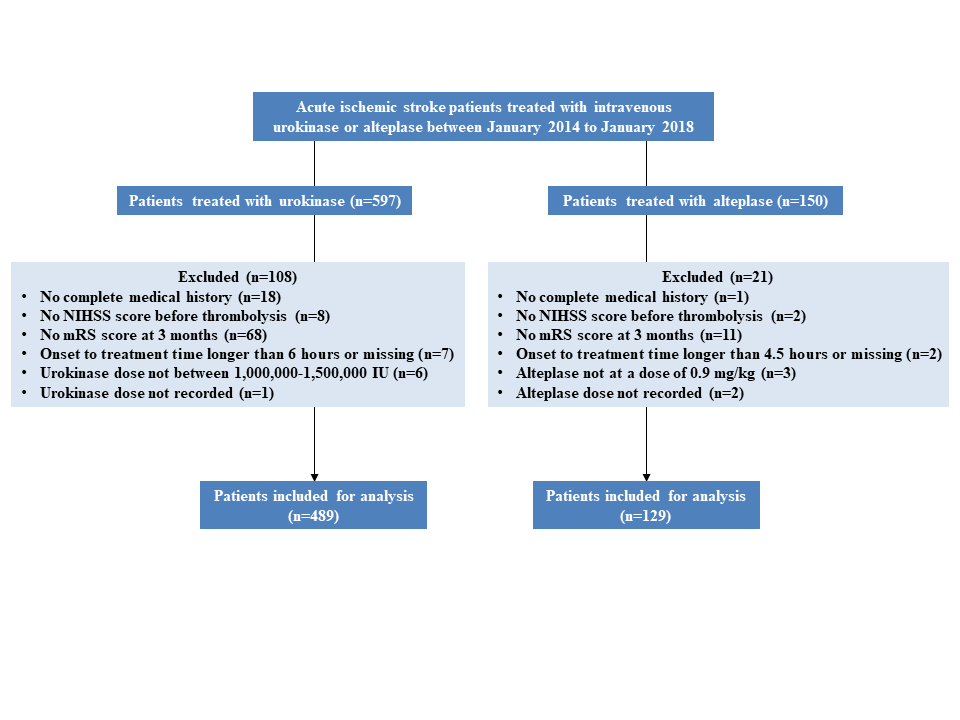


**Supplementary Figure 1.** **Study histogram.** NIHSS, National Institute of Health Stroke Scale; mRS, modified Rankin Scale.
